# Targeting blood thrombogenicity precipitates atherothrombotic events in a mouse model of plaque destabilization

**DOI:** 10.1038/srep10225

**Published:** 2015-05-11

**Authors:** Xiaoling Liu, Mei Ni, Lianyue Ma, Jianmin Yang, Lin Wang, Fangfang Liu, Mei Dong, Xiaoyan Yang, Mei Zhang, Huixia Lu, Jingjing Wang, Cheng Zhang, Fan Jiang, Yun Zhang

**Affiliations:** 1 Key Laboratory of Cardiovascular Remodeling and Function Research, Chinese Ministry of Education and Chinese Ministry of Health, and The State and Shandong Province Joint Key Laboratory of Translational Cardiovascular Medicine, Qilu Hospital, School of Medicine, Shandong University, Jinan, Shandong, China

## Abstract

Although some features of plaque instability can be observed in genetically modified mouse models, atherothrombosis induction in mice has been attested to be difficult. We sought to test the hypothesis that alterations in blood thrombogenicity might have an essential role in the development of atherothrombosis in ApoE−/− mice. In a mouse model of plaque destabilization established in our laboratory, we targeted blood thrombogenicity by systemically overexpressing murine prothrombin via adenovirus-mediated gene transfer. Systemic overexpression of prothrombin increased blood thrombogenicity, and remarkably, precipitated atherothrombotic events in 70% of the animals. The affected plaques displayed features of culprit lesions as seen in human coronary arteries, including fibrous cap disruption, luminal thrombosis, and plaque hemorrhage. Treatment with aspirin and clopidogrel substantially reduced the incidence of atherothrombosis in this model. Mechanistically, increased inflammation, apoptosis and upregulation of metalloproteinases contributed to the development of plaque destabilization and atherothrombosis. As conclusions, targeting blood thrombogenicity in mice can faithfully reproduce the process of atherothrombosis as occurring in human coronary vessels. Our results suggest that blood-plaque interactions are critical in the development of atherothrombosis in mice, substantiating the argument that changes in blood coagulation status may have a determinant role in the onset of acute coronary syndrome.

Atherosclerotic plaque disruption and subsequent luminal thrombosis (atherothrombosis) are responsible for most acute coronary syndromes (ACS) and sudden cardiac deaths^1^. Postmortem pathological studies suggested that plaques with a thin fibrous cap, a large necrotic core, sparse smooth muscle cells and collagen within the cap, and infiltration of macrophages and lymphocytes, are likely to be unstable^2^. Apart from pathological changes of the plaque, increased thrombogenicity of blood (the so called “vulnerable blood”) is also thought to have an indispensable role in the pathogenesis of ACS^2^. Accordingly, an animal model that can mimic the pathological plaque-blood interactions and faithfully reproduce the process of atherothrombosis as occurring in human coronary vessels will be essential for understanding the pathophysiological mechanisms of ACS and discovery of novel diagnostic and interventional strategies.

Genetically modified mice, for example apolipoprotein(E)–deficient (ApoE−/−) and low-density lipoprotein receptor knockout mice, are widely used to study atherogenesis. Several approaches to inducing plaque destabilization have been reported in mice, such as overexpression of p53, matrix metalloproteinase (MMP)-9 or urokinase^3^,^4^,^5^,^6^. Although features of plaque instability were observed in these mice, atherothrombotic events could not be consistently reproduced in these mice. Our laboratory has developed a mouse model of plaque destabilization by combining perivascular collar placement in the common carotid artery and stress exposure^7^. We have observed that in more than half of the mice treated with stress, the carotid plaques displayed features of destabilization such as decreased fibrous cap thickness, increased lipid contents and aggravated inflammation. However, consistent with other reported mouse models of plaque destabilization, typical atherothrombosis was rare. Recently, Chen *et al* applied a tandem stenosis to the carotid artery in ApoE−/− mouse and observed characteristics of plaque instability such as decreased collagen contents, increased inflammation, infiltration of macrophages and lymphocytes, and intra-plaque neovascularization^8^. Unfortunately, mainly intra-plaque hemorrhage, but not plaque disruption with concomitant thrombus, was observed in this model.

The mechanisms of this ‘atherothrombosis-resistance’ phenomenon in mice are poorly understood^2^,^9^. In human studies, it has been found that many silent plaque disruptions were detected without acute coronary events, suggesting that blood-plaque interactions may have an essential role in precipitation of the coronary atherothrombosis^10^. However, the importance of “vulnerable blood” in causing atherothrombosis has not been substantiated by direct experimental evidence. Interestingly, Welch *et al.* reported that atherothrombosis was observed in about 20% of ApoE/Npc1 double knockout mice, which might be related to increased thrombin generation and platelet activation due to Npc1 deficiency^11^. In light of these findings, we hypothesized that alterations in blood thrombogenicity may be critical in the development of atherothrombosis in mouse models of plaque destabilization. To test this hypothesis, in this study, we targeted blood thrombogenicity by systemically overexpressing murine prothrombin via adenovirus-mediated gene transfer in a mouse model of plaque destabilization established by stress-exposure, as shown in Fig. 1.

## Results

### Validation of the stress model of plaque destabilization

**Figure 1:**
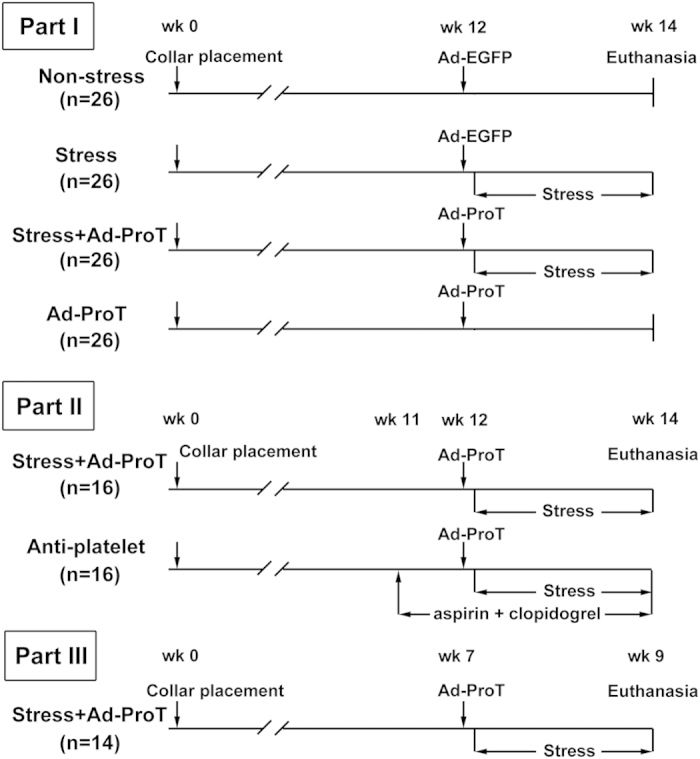
Flow charts showing the experimental protocol of *in vivo* studies. Ad-ProT, adenovirus expressing murine prothrombin; Ad-EGFP, adenovirus expressing EGFP.

In a mouse model of plaque destabilization established in our laboratory, we targeted blood thrombogenicity by systemically overexpressing murine prothrombin via adenovirus-mediated gene transfer (Fig. 1). Consistent with our previous study, stress stimulation induced a systemic stress response as evidenced by the increased plasma corticosterone levels and transient high blood pressure (Supplementary Table S1). Body weight was less in mice with stress stimulation than without, whereas the plasma lipid levels were not significantly different among four groups of mice (Supplementary Table S2).

**Figure 2:**
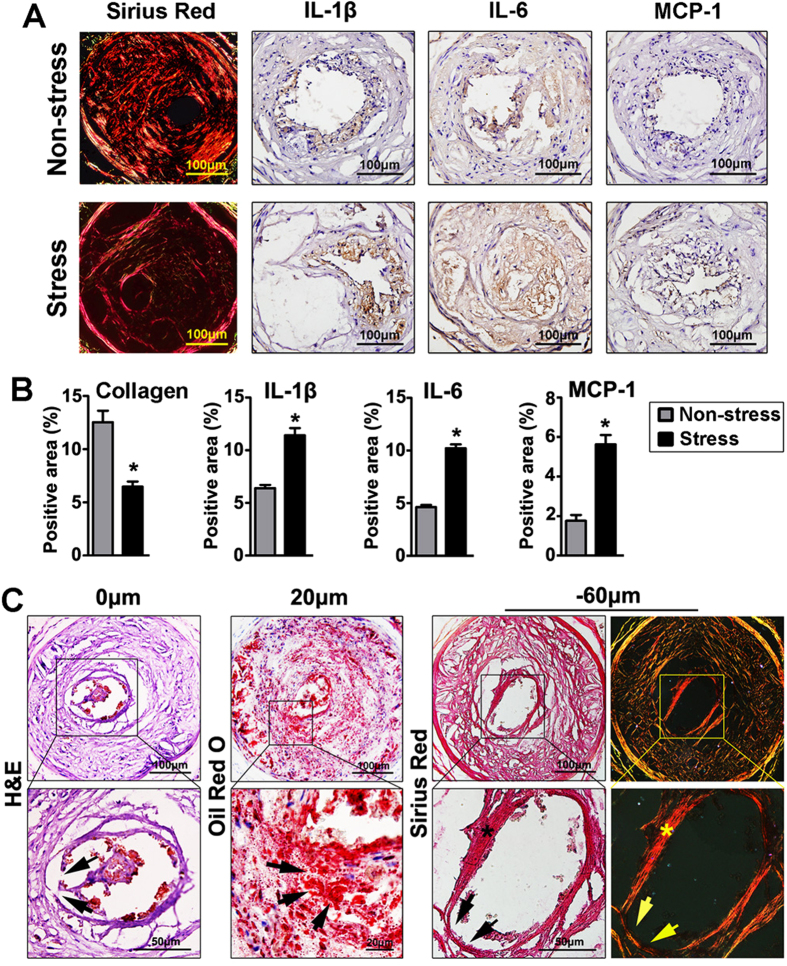
Stress-induced plaque destabilization in ApoE−/− mice. (**A**) The collagen examined by picrosirius red staining and expression of monocyte chemoattractant protein 1 (MCP-1), interleukin (IL)-1β and IL-6 measured by immunohistochemistry (brown) in plaques of the non-stress and stress groups. (**B**) Quantitative analysis of positive area of collagen, MCP-1, IL-1β and IL-6 in plaques. Data were mean ± SEM. * *P* < 0.05 *vs.* non-stress group, *n* = 14 for each group. (**C**) Representative images of plaque disruption. Fibrous cap disruption was revealed by H&E staining (left panel, arrow indicated the site of discontinuity of the fibrous cap). Middle panel was a section obtained 20 μm upstream of the previous section, which was stained with oil red O for lipids (red), showing that the plaque disruption occurred adjacent to a lipid-rich necrotic core (arrows). Right panels were sections obtained 60 μm downstream of the first section, which were stained with picrosirius red, showing the thin cap of the shoulder (arrow) compared to the other regions of the plaque (*).

Collar placement for 14 weeks induced advanced plaque in the right carotid artery of all mice, but no plaque was observed in non-collared left carotid arteries (Supplementary Fig. S1). Similar to our previous study, stress triggered plaque destabilization in the carotid artery, as evidenced by the decreased collagen content and enhanced inflammation (Fig. 2A,B). Pathological examination revealed 2 (14%) cases of plaque disruption in the stressed mice (Fig. 2C). In contrast, no plaque disruption was identified in the non-stressed group of mice (n = 14). By analyzing serial sections from the disrupted plaque, we found that the plaque shoulder area displayed features of vulnerability (thin fibrous cap and a focal lipid-rich necrotic area) (Fig. 2C). However, despite the presence of plaque destabilization in the stress group, atherothrombosis was still infrequent (14%) in these mice (Table 1).

### Prothrombin overexpression increased blood thrombogenicity in mice

To test the hypothesis that alterations in blood thrombogenicity are critical in the development of atherothrombosis in mice, murine prothrombin was overexpressed by systemic adenovirus transfection *via* tail vein injection. As shown in Supplementary Fig. S2, following adenovirus injection, most of the viruses were mainly enriched in the liver, and could be detected in the spleen, kidney, lung and plaques. To clarify the functional effects of prothrombin overexpression on coagulation, the concentration and activity of prothrombin as well as prothrombin time were examined in platelet-poor plasma. As shown in Table 2, prothrombin concentration and activity in plasma were increased whereas prothrombin time was decreased significantly after Ad-ProT transfection. In contrast, activities of factors VII and VIII, and the activated partial thromboplastin time (APTT) were not significantly altered by Ad-ProT transfection. In addition, platelet activation was measured with flow cytometry using CD62P as a marker, and it was found that CD62P-high platelets were increased by prothrombin overexpression (Table 2).

### Prothrombin overexpression precipitated acute atherothrombotic events

**Figure 3:**
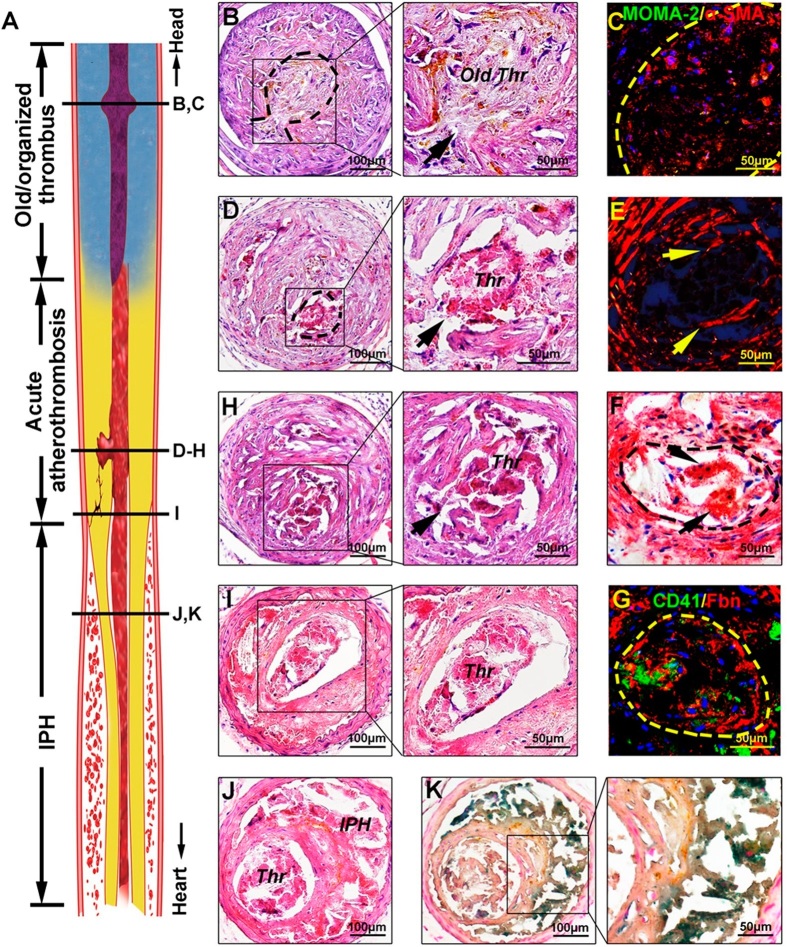
Stress+Ad-ProT-induced atherothrombosis and intra-plaque hemorrhage in the carotid artery. (**A**) A longitudinal sectioned schematic diagram of a carotid artery showing old/organized thrombus, acute plaque disruption and thrombosis, and intraplaque hemorrhage (IPH). (**B**) A partially organized old thrombus (dashed line) stemming from the aperture of the disrupted plaque (arrow) and causing total occlusion of the lumen. (**C**) The organized thrombus (dashed line) displayed presence of infiltrating cells mainly positive for smooth muscle α-actin (α-SMA, red). (**D,H**) Acute plaque disruption (arrow) and luminal thrombus formation (dashed line) revealed by H&E staining. (**E**) Fibrous cap disruption (arrows) revealed by picrosirius red staining. (**F-G**) Acute luminal thrombus (dashed line) contained foam cell debris revealed by oil red O staining (arrows in F), platelets (identified by CD41, green in G) and fibrin (Fbn, red in G). (**I**) The body of the same thrombus as in (**D-H**). (**J**) Intra-plaque hemorrhage coexisted with thrombus. (**K**) Perl’s iron staining showed the blood pool was positive for ferric iron (blue color), indicating the presence of degraded hemoglobin. *Thr*, thrombus; *IPH*, intraplaque hemorrhage.

In stressed prothrombin-overexpressing mice, we identified acute carotid plaque disruption associated with luminal thrombi (atherothrombosis, Fig. 3) in 71% (10 of 14) of the mice. Acute thrombi contained plaque debris, aggregated platelets and sheets of fibrin (Fig. 3D–3H). Some plaques were associated with partially organized old thrombi, which were characterized by hyaline degeneration, cell infiltration (mainly by smooth muscle cells) and brownish iron deposits (Fig. 3B,C). Of note, we found that 5 of the 10 acute atherothrombotic lesions also colocalized with old, partially organized thrombi buried in the same plaque (Fig. 3), indicating that these plaques had disrupted for more than once, an important feature of culprit lesions in human coronary arteries^12^.

Furthermore, we observed extensive intraplaque hemorrhage in 4 mice (29%, Table 1), which could be identified macroscopically as hematoma-like lesions in the vessel wall and microscopically as red blood cell accumulation within the plaque (Supplementary Fig. S3). Histological examination of the serial sections demonstrated that in 3 of the 4 mice, intraplaque hemorrhage occurred at or around the region of plaque disruption (Fig. 3J,K, Supplementary Fig. S3), and there were no evidence of intraplaque neovascularization in these lesions. Thus, it is likely that intraplaque hemorrhage observed in these mice was mainly due to plaque disruption and originated from the lumen via the aperture of disrupted plaque (Supplementary Fig. S3C and S3D). In the last mouse with intraplaque hemorrhage, neither plaque disruption nor plaque neovascularization was observed and the cause of intraplaque hemorrage was obscure. In most of these mice, intraplaque hemorrhage resulted in a large pool of red blood cells, dissection of the vessel wall and dilapidated plaque (Supplementary Fig. S3E-S3G).

To eliminate the possibility that these atherothrombotic lesions were results of spontaneous thrombus formation due to prothrombin overexpression, we looked at the liver, lung, spleen and kidney by gross and microscopic (H&E staining) examination but did not find any evidence of thrombosis and tissue infarction (data not shown) in these organs, which indicated that the thrombosis observed in carotid arteries was a result of plaque disruption. To examine the reproducibility of these results, we repeated the same experiment in another stress+Ad-ProT group (n = 16, Part II *in vivo* study) with the same dose of Ad-ProT injection and stress exposure, which showed that the rates of atherothrombosis and intraplaque hemorrhage in the collared carotid artery were 69% (11 out of 16) and 25% (4 out of 16) respectively. The pooled data were summarized in Table 1.

### Atherothrombosis requires vulnerabilities in both blood and plaque

**Figure 4:**
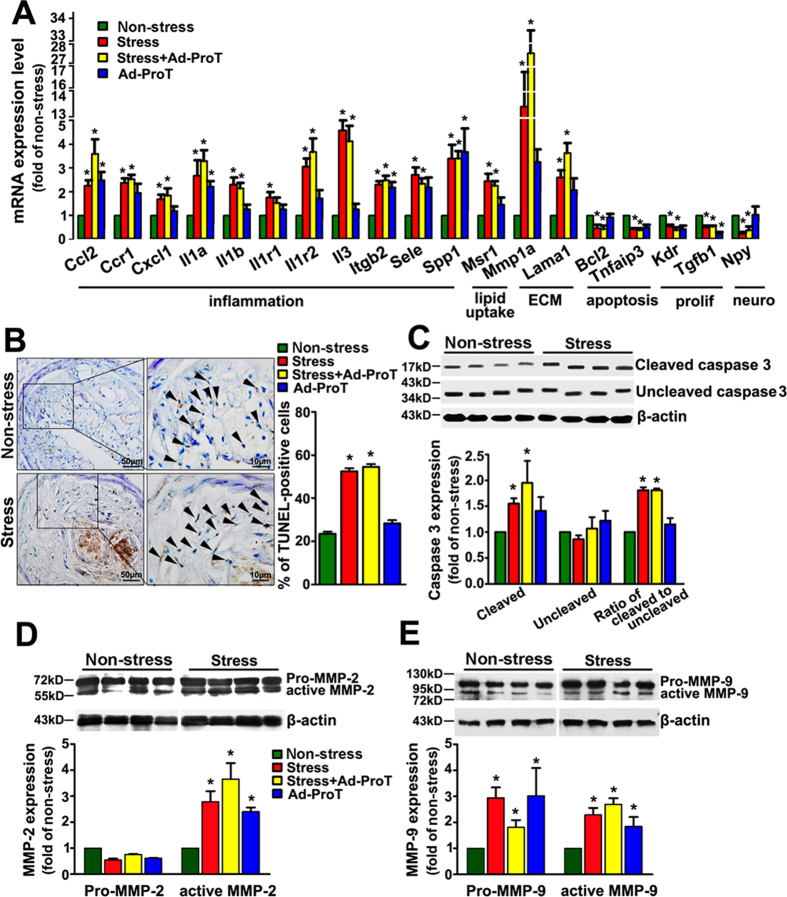
Effects of stress and/or prothrombin overexpression (Ad-ProT) on vascular inflammation, apoptosis and MMP expression. (**A**) Altered gene expressions in carotid plaques as detected by mouse atherosclerosis PCR array (n = 6 for each group). * *P* < 0.05, vs. non-stress group. Genes were grouped according to their cellular functions. ECM: extracellular molecules; prolif: cell proliferation; neuro: neurotransmission. See Supplementary Table S3 for specific gene names. (**B**) Apoptosis in the carotid plaque assessed by TUNEL labeling. The images were representative examples from mice in the non-stress and stress groups respectively. Arrows indicated apoptotic cells. Quantitative data were shown on the right (mean ± SEM; * *P* < 0.05 *vs.* non-stress group, n = 14 for each group). (**C, D, E**) Western blots showing caspase 3 activation (cleaved to uncleaved caspase 3 ratio), and pro- and active MMP-2 and MMP-9 expression in the carotid artery. Bar graphs at the bottom were quantitative densitometry data expressed as fold of non-stress mice. * *P* < 0.05 *vs.* non-stress group, n = 4 for each group.

In contrast to the high incidence of atherothrombosis in stress+Ad-ProT mice, Ad-ProT alone mice displayed atherothrombosis only in 3 out of 14 carotid plaques (21%, Table 1). This indicated that atherothrombosis was a result of the synergistic effects of stress and prothrombin overexpression. To further clarify the mechanisms involved in carotid plaque destabilization and atherothrombosis, we analyzed expression of atherosclerosis-related genes in carotid arteries using Superarray Atherosclerosis PCR arrays. This commercial array included 84 genes belonging to several subsets of gene families that have been implicated in atherogenesis, including inflammatory responses, apoptosis, adhesion molecules, lipid metabolism, cell growth, and transcription regulators. The expression profile of all examined genes was shown in supplementary Table S3. We found that regardless of the presence of prothrombin overexpression, stress significantly upregulated expression of 11 genes involved in inflammation, 1 gene involved in lipid uptake and 1 gene of matrix metalloproteinase, and downregulated 2 anti-apoptotic genes and 2 genes involved in proliferation (Fig. 4A). To clarify whether plaque destabilization was associated with excessive cell apoptosis, we performed TUNEL labeling experiments and immunostainning for cleaved caspase 3. We demonstrated that the number of apoptotic cells in the plaque and the level of cleaved caspase-3 were significantly increased in stressed mice (Fig. 4B,C and Supplementary Fig. 4). In addition, we found that the expression of both active MMP-2 and MMP-9 was increased in vessels from stressed mice (Fig. 4D,E). In contrast, prothrombin overexpression had only modest effects on inflammation and apoptosis as compared to the effects of stress, although prothrombin increased MMP expression (Fig. 4). Together with the low incidence of atherothrombosis in mice with prothrombin overexpression alone, it is suggested that prothrombin *per se* is unlikely to have a major role in modulating plaque stability in this model. Thus, we demonstrated that atherothrombosis was precipitated by enhanced blood thrombogenicity by prothrombin overexpression on the basis of plaque destabilization induced by stress exposure in this mouse model.

### Atherothrombosis was prevented by anti-platelet therapy

Anti-platelet therapy is effective in preventing coronary atherothrombotic events in humans. In order to confirm that our mouse model is relevant to human disease, we tested whether anti-platelet drugs used in clinical practice could prevent the occurrence of atherothrombosis. We treated the stress+Ad-ProT mice with aspirin (5 mg/kg/day) and clopidogrel (25 mg/kg/day) by oral administration for the last 3 weeks. We found that while the platelet activation was significantly depressed (3.13 ± 0.47% vs. 15.90 ± 1.90%, *P *< 0.05, n = 8 for each group), the incidence of atherothrombosis was significantly reduced to 19% (3 out of 16) (Table 1, *P *< 0.05). In contrast, in line with a previous study^13^, aspirin and clopidogrel co-treatment had no significant effect on the pathological features of the carotid plaque (data not shown).

### Onset of atherothrombosis was plaque size-dependent

**Figure 5:**
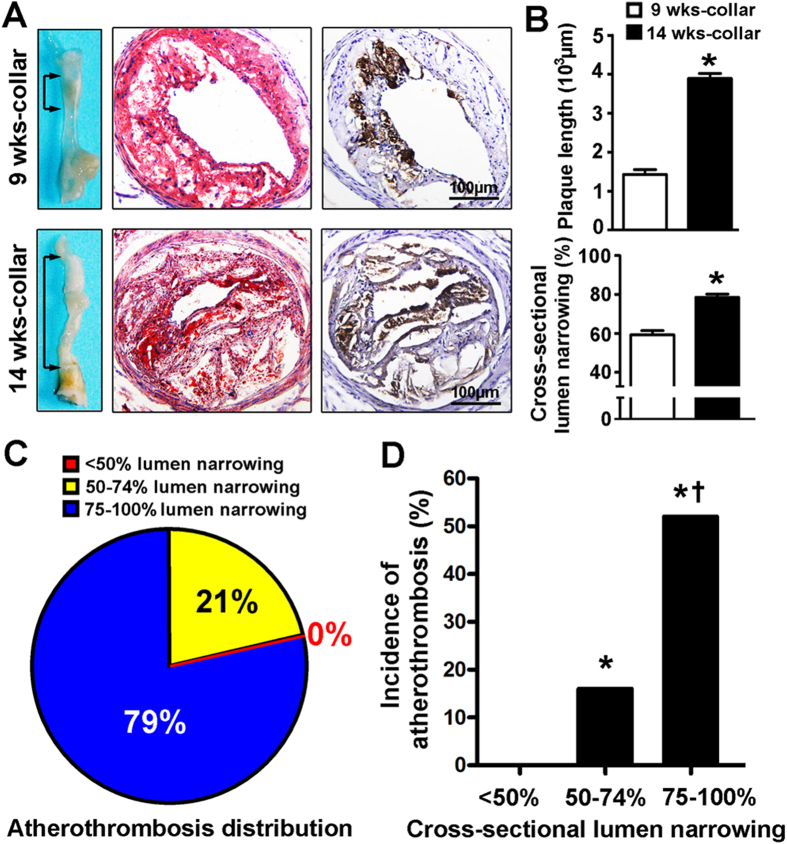
Relationship between atherothrombosis and degree of lumen stenosis. (**A**) Plaque burden of carotid lesions in stress+Ad-ProT mice with collar treatment for 14 and 9 weeks. The left panels show the gross view of carotid plaques (arrows) after collar placement for 9 weeks and 14 weeks respectively. Lipids were stained with oil red O (middle panel) and macrophages were immuno-stained with MOMA-2 antibody (right panel, brown color). (**B**) Quantitative analysis of the plaque length and cross-sectional lumen narrowing in carotid arteries. Plaque length was calculated by multiplying the thickness (5 μm for each) of all cross sections with atherosclerotic lesions. *P < 0.05 vs. 9 weeks group (n = 14–30). (**C**) Analysis of plaque size distribution amongst all atherothrombosed plaques. Pooled data from the non-stress, stress, stress + Ad-ProT (9 and 14 weeks) and Ad-ProT groups of mice (n = 28). (**C**) Incidence of atherothrombosis in carotid plaques with different degrees of lumen narrowing. Pooled data from the non-stress, stress, stress + Ad-ProT (9 and 14 weeks) and Ad-ProT groups of mice. For plaques with <50%, 50–74% and 75–100% luminal narrowing, n = 6, 38 and 42 respectively. *P < 0.05 vs. <50% luminal narrowing, ^†^*P* < 0.05 vs. 50–74% luminal narrowing.

To determine whether atherothrombosis was inducible in less severe lesions, Ad-ProT and stress treatment was given to mice with a shorter period (i.e. 9 weeks) of collar implantation. Compared with 14-week samples, 9-week samples showed much smaller plaque burden and lumen narrowing (Fig. 5A,B). In these 9-week samples, only 2 (14%) mice exhibited atherothrombosis, including 1 mouse with fresh disruption and 1 with old thrombus.

We analyzed plaque size distribution amongst all atherothrombosis-complicated plaques, and revealed that 79% of atherothrombosis occurred in plaques with 75%–100% cross-sectional narrowing and 21% in plaques with 50%–74% cross-sectional narrowing. In contrast, no atherothrombosis was observed in plaques with <50% cross-sectional narrowing (Fig. 5C). Likewise, there was a stepwise increase in the incidence of plaque disruption and atherothrombosis with increased cross-sectional lumen narrowing, which was consistent with the findings in human beings (Fig. 5D)^14^.

### Plaque complications in other arteries

Pathological studies of the plaque at the aortic root, coronary artery, innominate artery and abdominal aorta were performed and no signs of plaque disruption and/or atherothrombosis were found. This may be partly due to the lower degree of stenosis at these sites (<50% cross-sectional lumen narrowing, Supplementary Fig. S5) as compared to the collared carotid artery (~80% narrowing).

## Discussion

The major finding of the present study was that targeting blood thrombogenicity by overexpressing murine prothrombin, in an ApoE−/− mouse-based, stress-induced plaque destabilization model, successfully precipitated atherothrombosis in about 70% of the animals. This result advanced previous studies, in which different approaches were applied to manipulate the plaque stability but the overall incidence of typical atherothrombosis was considerably low^3^,^4^,^8^,^11^,^15^. Pathohistological studies revealed that many of these atherothrombotic lesions were associated with both fresh and older or organized thrombi, indicating that they had undergone plaque disruption for more than once, a feature similar to human coronary lesions^16^. Moreover, we showed that dual anti-platelet drug therapy substantially reduced the incidence of plaque disruption and atherothrombosis, indicating that this model may be potentially useful in preclinical drug developments.

The concept of “vulnerable blood” has been proposed for some time^10^. This is mainly based on human studies indicating that ACS are associated with prothrombotic conditions of the blood, caused by increased platelet activation, dysregulated coagulation factors, and/or decreased anticoagulation factors and endogenous fibrinolysis activity^17^. In this study, we demonstrated that a short term overexpression of prothrombin leading to increased thrombogenic potential of the blood effectively precipitated atherothrombotic events in the presence of plaque destabilization, highlighting the possibility that transient unbalance between coagulation and anticoagulation activities may have a critical role in triggering ACS in patients. Our study is also in agreement with previous studies showing that the high incidence of spontaneous atherothrombosis observed in ApoE/Npc1 double knockout mice is likely to be, at least partially, attributable to increased thrombin generation and platelet activation secondary to Npc1 deficiency^11^.

As a systemic response, stress-induced neuronal and humoral reactions may affect plaque stability and cardiovascular events by modulating multiple pathways including hemodynamic stress, angiotensin II stimulation, increased vascular tone, and enhanced inflammatory reaction^8^,^18^. Consistent with our previous results, the present PCR array and western blotting experiments confirmed that stress-induced plaque destabilization was associated with increased vascular inflammation, apoptosis and MMP upregulation. However, we noted that stress alone did not result in reproducible atherothrombosis, further supporting the critical role of blood-plaque interactions in triggering atherothrombotic events in mice. Apart from effects on blood coagulation, the increased (pro)thrombin may also affect plaque stability by modulating gene expression and function of vascular cells^19^,^20^,^21^. However, our results did not show remarkable effects of prothrombin overexpression alone on vascular inflammation and cell apoptosis in plaques. Together with the observation that prothrombin alone did not cause typical atherothrombosis, it was unlikely that prothrombin had a major impact on plaque stability as did stress treatment. Likewise, aspirin and clopidogrel treatment had little effects on the size and composition of the carotid plaques, suggesting that the anti-atherothrombotic effect of aspirin and clopidogrel was not mediated by direct modification of the plaque *per se*^13^, but likely to be associated with reduced “vulnerability of the blood”.

Accumulating evidence suggests that the occurrence of ACS is commonly associated with an increased plaque burden in major coronary arteries^14^,^22^. The PROSPECT trial showed that a large plaque burden (≥70%) and a small luminal area (<4 mm^2^), as determined by intravascular ultrasound, were independent predictors of future cardiovascular events^14^. In this study, we found that plaque disruption and luminal thrombi were mainly present in lesions with >75% cross-sectional narrowing. Furthermore, atherothrombosis was exclusively induced in collar-induced carotid plaques, whereas lesions in the aortic root, coronary artery, innominate artery and abdominal aorta were found to be free of atherothrombosis. The mechanisms of the different sensitivity of plaques to atherothrombosis were not completely understood, but this difference may be partially explained by the lower plaque burden in other arteries as compared with the advanced plaques in the carotid artery.

We created this atherothrombosis model by enhancing blood thrombogenicity in an established mouse model of stress-induced plaque destabilization. The use of adenoviral vectors for gene delivery of prothrombin was associated with some limitations, such as the transient nature of expression of the ectopic gene and the difficulty in precise control of the gene expression level. Hence, additional studies are warranted to develop an inducible transgenic system, which can stably increase the blood thrombogenicity to a level that is comparable to that in ACS patients.

**Figure 6:**
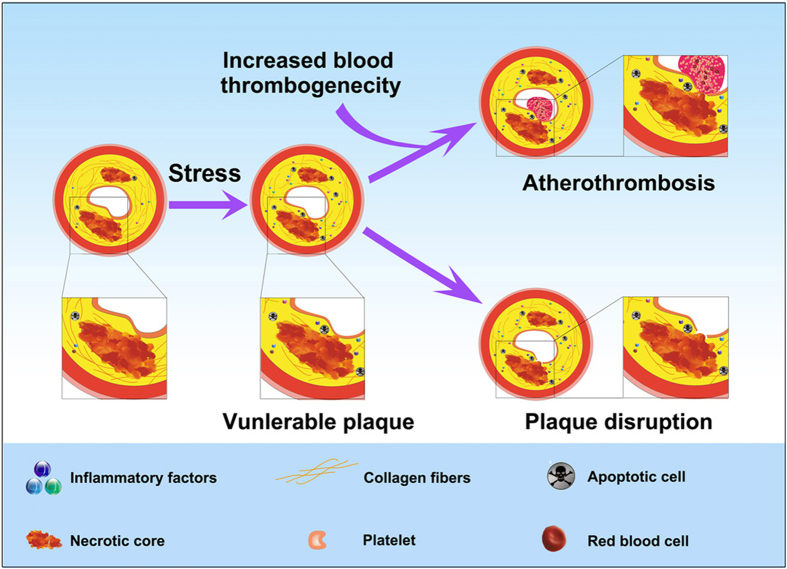
A cartoon showing the mechanisms of plaque destabilization and atherothrombosis in the present mouse model. Stress may turn a stable plaque into a vulnerable plaque phenotype, leading to plaque disruption. Plaque disruption and concomitant increase in thrombogenicity may precipitate atherothrombosis events. Our results support the notion that blood-plaque interactions may have a determinant role in the onset of acute coronary syndromes.

In summary, we demonstrated that prothrombin overexpression in a mouse model of plaque destabilization consistently reproduced the process of occlusive atherothrombosis, which was similar to those found in human coronary arteries causing ACS. Our data support the argument that changes in blood coagulation status may have a determinant role in the onset of ACS (Fig. 6).

## Methods

An expanded methods section is provided in the online Data Supplement.

### Construction of a recombinant adenovirus expressing prothrombin

A cDNA clone of murine prothrombin (purchased from OriGene, catalogue # MC205384) was subcloned into pAd/CMV/V5-Dest vector (Invitrogen, USA) (Ad-ProT) for adenovirus packaging in 293A cells. The adenovirus expressing EGFP alone (Ad-EGFP) was used as control.

### Animal experiments

A total of 150 ApoE−/− mice on a C57BL/6 background were maintained on a high-fat diet and a perivascular collar was implanted around the right carotid artery to accelerate atherosclerotic lesion formation^7^,^23^. The *in vivo* study design was shown in Fig. 1.

Part I: One hundred and four mice were randomly divided into four groups (n = 26 in each group, 14 for histological analysis and 12 for molecular biological analysis): non-stress, stress, stress + Ad-ProT and Ad-ProT. Ad-ProT (5 × 10^9^ pfu) was injected via the tail vein 12 weeks after collar placement, and Ad-EGFP at the same dose was injected in other groups as a control. Stress stimulation was applied by repeated restraint in a well ventilated narrow space (50 ml, 6 hrs per day, 5 days per week for 2 weeks) as described previously^24^.

Part II: To examine the reproducibility of the model of atherothrombosis, and examine the effects of anti-platelet drugs, thirty-two mice were divided randomly into two groups (n = 16 each): a repeated stress+Ad-ProT group and stress+Ad-ProT+anti-platelet treatment group. Anti-platelet treatment was done by oral administration of aspirin at 5 mg/kg/day and clopidogrel at 25 mg/kg/day during the last 3 weeks^13^.

Part III: To determine whether atherothrombosis can be induced in less severe lesions, Ad-ProT and stress treatment was performed in 14 mice with a shorter period of collar treatment (i.e. 9 weeks).

At the end of the experiments, all mice were euthanized with an injection of overdose pentobarbital (50 mg/kg), blood was drawn from the inferior vena cava into tubes containing trisodium citrate, and tissues including heart, aorta, carotid arteries, lung, liver, spleen and kidneys were dissected and preserved for histological and molecular biological analysis.

### Ethics statement

The animal experiment was approved by the Animal Care Committee of Shandong University. The methods were carried out in accordance with the approved guidelines: the Animal Management Rules of the Chinese Ministry of Health (Document No. 55, 2001).

### Histological and morphological analysis

Segments of the common carotid artery with plaques were embedded in OCT compound (Tissue-Tek, Sakura Finetek) and continuous transverse cryosections of 5 μm in thickness were cut, which covered the whole length of the plaque. Generally this yielded 800–1000 sections (on 200–250 slides) per animal. The every 5th slide was stained with hematoxylin and eosin (H&E) for morphometric measurements. Special staining was performed with oil red O for lipids, picrosirius red for collagen, Perl’s stain for ferric iron, and immunohistochemical staining with specific antibodies. Apoptosis was assessed with a terminal deoxynucleotidyl transferase end-labeling (TUNEL) kit (from Millipore, USA). Plaque disruption was defined as presence of a structural defect in the fibrous cap that separates a necrotic core of an atherosclerotic plaque from the lumen, resulting in exposure of the necrotic core to the blood via the gap in the cap^25^. Atherothrombosis was defined as plaque disruption with superimposed thrombus enriched with platelets and fibrin^26^. Intraplaque hemorrhage was ascertained by the existence of abundant red blood cells within the plaque^27^.

The lung, liver, spleen and kidney samples were embedded in paraffin, and serial 5 μm-thick cross sections were prepared at an interval of 50 μm. Normally we obtained 100–400 sections per organ, and surveyed all of them for signs of thrombosis. Histological and morphometric analyses were performed by three independent researchers blindly.

### Western blot

Total proteins from artery samples were separated by 10% or 15% SDS-PAGE and transferred to Immobilon-P (PVDF) membranes. The membrane was blocked with 5% non-fat milk and then incubated with primary antibodies at 4 °C overnight. The blots were developed with enhanced chemiluminescence reagent (Millipore).

### PCR array analysis

Total RNA was extracted from the region proximal to the carotid collar and reversely transcribed to cDNA. Expression levels of a set of 84 atherosclerosis-related genes were examined using Atherosclerosis RT2 Profiler PCR Arrays (Qiagen-SABiosciences, USA) according to the manufacture’s protocol. The complete list of the genes analyzed is available online at http://www.sabiosciences.com/rt_pcr_product/HTML/PAMM-038A.html. Data analysis was performed with ΔΔCt-based fold-change calculations using a software package provided by the manufacturer.

### Blood coagulation test

The concentration of prothrombin in platelet-poor plasma was quantified using a commercial ELISA kit (ASSAYPRO, USA). The activity of coagulant factor II (prothrombin), VII and VIII, and the parameters of prothrombin time (PT) and activated partial thromboplastin time (APTT) were measured using clotting assays with reagents used for human clotting factor determination in an automated coagulation analyzer (STA-R Evolution, Stago, France).

### Flow cytometry for platelet activation assessment

CD41 was used as a marker for platelets. CD62P was used as a marker for platelet activation. Flow cytometry was performed using FACS CALIBUR cytometer (BD Biosciences, USA).

### Plasma corticosterone level

The plasma corticosterone levels were quantified using a Corticosterone Enzyme Immunoassay Kit (Assay Designs, AnnArbor, MI, USA).

### Plasma lipid profile

The plasma levels of total cholesterol, triglycerides, low-density lipoprotein cholesterol, and high-density lipoprotein cholesterol were measured using commercial kits (Roche Diagnostics, Indianapolis, IN).

### Statistical analysis

Quantitative values are expressed as mean ± standard error of the mean (SEM) and analyzed by unpaired *t*-test or one-way ANOVA as appropriate. Qualitative data were analyzed by chi-square test. A level of *P* < 0.05 was considered significant.

## Author Contributions

X.L., M.N. and L.M. designed all of the experiments, performed *in vivo* studies and wrote the manuscript; J.Y., L.W., F.L., M.D., X.Y., M.Z. and H.L. performed *in vitro* assays; J.W. and C.Z. were involved in data interpretation and statistical analysis. F.J. and Y.Z. conceived the study, revised and approved the manuscript. All authors reviewed the manuscript.

## Additional Information

**How to cite this article**: Liu, X. *et al.* Targeting blood thrombogenicity precipitates atherothrombotic events in a mouse model of plaque destabilization. *Sci. Rep.* **5**, 10225; doi: 10.1038/srep10225 (2015).

## Supplementary Material

Supplementary Information

## Figures and Tables

**Table 1:** **Incidence of atherothrombosis and intraplaque hemorrhage within plaque in three *in vivo* studies**.

**Groups**	**Cross-sectional lumen narrowing (%)**	**Incidence of plaque ruptures and thrombosis**				**Length of thrombus (μm)**	**Incidence of intraplaque hemorrhage**	**Incidence of combined thrombi and hemorrhage**
		**Total (%)**	**Acute**	**Older**	**Both**			
***Part I in vivo***								
Non-stress (n = 14)	77.0 ± 2.1	0/14 (0%)	—	—	—	—	0/14 (0%)	—
Stress (n = 14)	77.9 ± 2.5	2/14(14%)	2/14	1/14	1/14	465 ± 79	0/14 (0%)	—
Stress+Ad-ProT (n = 14)	79.3 ± 2.1	10/14 (70%)*	10/14	5/14	5/14	1042 ± 178	4/14 (29%)*	3/14*
Ad-Pro T(n = 14)	79.3 ± 2.8	3/14 (21%)	3/14	1/14	1/14	469 ± 112	3/14 (21%)	2/14
								
***Part II in vivo***								
Stress+Ad-ProT (n = 16)	79.6 ± 1.4	11/16 (69%)*	7/16	8/16	4/16	807 ± 221	4/16 (25%)*	4/14*
Anti-platelet treatment (n = 16)	77.0 ± 2.3	3/16 (19%)^†^	3/16	1/16	1/16	446 ± 409	0/16 (0%)^†^	—
								
***Part III in vivo***								
Stress+Ad-ProT-9 wks (n = 14)	60.5 ± 2.4^‡^	2/14 (14%)	1/14	1/14	0/14	443 ± 293	2/14 (14%)	2/14

Numerators are the number of animals with indicated plaque complications; the denominators are the total number of animals in the group. Continuous data are expressed as mean ± SEM. Length of thrombus was calculated by multiplying the thickness of cross sections with the number of total sections. *P < 0.05 *vs.* stress group; ^†^*P *< 0.05 *vs*. stress + Ad-ProT group; ^‡^*P* < 0.05 *vs.* stress + Ad-ProT mice with collar placement for 14 weeks. Ad-ProT, adenovirus expressing prothrombin.

**Table 2:** **Coagulation parameters in platelet-poor plasma**.

**Parameters**	**Non-stress (n** **=** **6)**	**Stress (n** **=** **6)**	**Stress+Ad-ProT (n** **=** **6)**	**Ad-ProT (n** **=** **6)**
Prothrombin concentration (ug/ml)	109.05 ± 7.56	104.95 ± 7.74	145.99 ± 9.02*^†^	143 ± 9.25*^†^
Prothrombin activity (%)	74.33 ± 4.67	73.17 ± 2.85	89.33 ± 2.44*^†^	91.00 ± 2.53*^†^
PT (s)	12.62 ± 0.29	12.73 ± 0.44	11.92 ± 0.13^†^	11.68 ± 0.07*^†^
Factor VII activity (%)	1300 ± 115	1156 ± 79	1168 ± 74	1220 ± 39
Factor VIII activity (%)	513 ± 32	524 ± 32	518 ± 28	484 ± 19
APTT (s)	62.10 ± 7.06	61.30 ± 5.87	55.73 ± 3.46	57.17 ± 4.36
Platelet activation (%)	3.19 ± 0.53	10.08 ± 0.96*	15.90 ± 1.90*	10.84 ± 1.31*

PT, prothrombin time; APTT, activated partial thromboplastin time. ^*^P < 0.05, vs. non-stress group; ^†^*P* < 0.05, vs. stress group.
