# Supplementary material for: Targeting blood thrombogenicity precipitates atherothrombotic events in a mouse model of plaque destabilization
Source: Sci Rep. 2015 May 11;5:10225. doi: 10.1038/srep10225 (PMC4426696; doi:10.1038/srep10225)
Supplement: Supplementary Information [file srep10225-s1.doc]

**Supplemental Data**

**Targeting blood thrombogenicity precipitates atherothrombotic events in a mouse model of plaque destabilization**

***By:***

Xiaoling Liu ^†^; Mei Ni ^†^; Lianyue Ma ^†^; Jianmin Yang; Lin Wang; Fangfang Liu; Mei Dong; Xiaoyan Yang; Mei Zhang; Huixia Lu; Jingjing Wang; Cheng Zhang; Fan Jiang*; and Yun Zhang*

***From:***

Key Laboratory of Cardiovascular Remodeling and Function Research, Chinese Ministry of Education and Chinese Ministry of Health, and the State-Province Co-cultivated Key Laboratory of Translational Cardiovascular Medicine, Qilu Hospital, School of Medicine, Shandong University, Jinan, Shandong, China

^†^ The three authors contributed equally to this study.

Address for correspondence: Yun Zhang, MD, PhD, FACC, FESC, FASE, E-mail: [**zhangyun@sdu.edu.cn**](mailto:zhangyun@sdu.edu.cn), TEL: +86-531-82169257, FAX: +86-531-86169356;

or Fan Jiang, PhD, E-mail: [**fjiang@sdu.edu.cn**](mailto:fjiang@sdu.edu.cn), TEL: +86-531-82169267, Qilu Hospital, Shandong University, 107 Wen Hua Xi Road, Jinan, Shandong 250012, China.

**Methods**

***Construction of a recombinant adenovirus expressing prothrombin***

A cDNA clone of murine prothrombin (OriGene, USA) was amplified by PCR and the PCR product was inserted into the pIRES2-EGFP vector. The prothrombin-IRES-EGFP construct, which contained an internal ribosome entry site allowing bicistronic expression of the target gene and EGFP as a marker, was then subcloned into pAd/CMV/V5-Dest vector (Invitrogen, USA) for adenovirus packaging in 293A cells. The recombinant virus (Ad-ProT) was purified and concentrated before *in vivo* administration. The adenovirus expressing EGFP alone (Ad-EGFP) was used as a control.

***Animal experimental protocol***

A total of 150 ApoE-/-mice on C57BL/6 background (male, 12 weeks old) were obtained from Peking University Animal Center (Beijing, China). Mice were fed on a high-fat diet containing 0.25% cholesterol and 15% cocoa butter under standardized lighting conditions (12h light -12h dark cycle) and temperature (21±1 °C). Atherosclerotic lesions were induced by placement of a silastic perivascular collar (3 mm in length and 0.3 mm in internal diameter) around the right carotid artery as described previously ^1^. These mice were then assigned to three parts of *in vivo* studies as shown in Figure 1.

Part I: To examine the effects of prothrombin and stress on advanced carotid plaques, one hundred and four ApoE-/-mice were randomly divided into four groups at the end of week 12: non-stress, stress, stress+Ad-ProT and Ad-ProT (n=26 for each group, 14 for histological analysis and 12 for molecular biological analysis). Mice in the stress+Ad-ProT and Ad-ProT groups received an intravenous injection of 5×10^9^ pfu of Ad-ProT, while others received an intravenous injection of 5×10^9^ pfu of Ad-EGFP. Two days after adenovirus injection, mice in the stress and stress+Ad-ProT groups were exposed to stress stimulation exerted by restraint in a well ventilated syringe (50 ml) for 6 hrs per day and 5 days per week, as described previously ^2^, for two weeks. To evaluate the acute and chronic effects of stress, systolic blood pressure (SBP), diastolic blood pressure (DBP), heart rate (HR) and plasma levels of corticosterone were measured 1 and 20 hours after the final stress stimulation ^2^. Hemodynamic parameters were measured using a noninvasive tail-cuff system (Softron BP-98A, Tokyo, Japan).

Part II: To examine the reproducibility of the model of atherothrombosis, and examine the effects of anti-platelet drugs, thirty-two mice were divided randomly into two groups (n=16 for histological analysis in each group) at the end of week 12: a repeated stress+Ad-ProT group and stress+Ad-ProT+anti-platelet treatment group. Antiplatelet treatment was done by oral administration of aspirin at 5 mg/kg/day and clopidogrel at 25 mg/kg/day during the last 3 weeks ^3^.

Part III: to determine whether atherothrombosis can be induced in less severe lesions, Ad-ProT and stress treatment was performed in 14 mice with a shorter period of collar placement (i.e. 9 weeks).

All mice underwent euthanasia with injection of overdose pentobarbital (50 mg/kg) at the end of experiment. Blood was drawn from the inferior vena cava into tubes containing trisodium citrate, and tissues including the heart, aorta, carotid arteries, lung, liver, spleen and kidney were dissected and preserved for histological and molecular biological analysis. The animal experimental protocol complied with the Animal Management Rules of the Chinese Ministry of Health (Document No. 55, 2001) and was approved by the Animal Care Committee of Shandong University.

***Histological and morphological analysis***

To observe the extent of adenovirus transfection, the liver, spleen, kidney, lung and carotid plaques were collected from 2 non-stress mice at day 8 and day 15 (the end of study) after adenovirus injection respectively. These samples were embedded in paraffin and serial 5 μm-thick paraffin sections were prepared at an interval of 50 μm. To avoid the confusion of autofluorescence, the expression of GFP was detected by immunohistochemical staining using specific anti-GFP antibody (1:500, Abcam, UK).

Atherosclerotic plaques were first identified in the aorta and its major branches with gross examination, and then embedded in OCT compound (Tissue-Tek, Sakura Finetek). Continuous transverse cryosections of 5 μm in thickness were cut, which covered the whole length of a given plaque. Generally this yielded 800-1000 sections (on 200-250 slides) per carotid plaque and ~ 100 sections (on ~ 20 slides) per plaque at other locations. The every 5th slide was stained with hematoxylin and eosin (H&E) for morphometric measurements. Special staining was performed with oil red O for lipids, picrosirius red for collagen and Perl’s stain for ferric iron. For immunohistochemical staining, the following antibodies were used: anti-macrophage (MOMA-2, 1:100, AbD Serotec, UK), anti-smooth muscle cell α-actin (α-SMA, 1:100, Abcam), anti-monocyte chemoattractant protein-1 (MCP-1) (1:50, Abcam), anti-interleukin (IL)-1β (1:100, Abcam), anti-IL-6 (1:100, Abcam), anti-fibrin (1:100, Abcam), anti-CD41 (1:100, Abcam), anti-CD31 (1:100, BD pharmingen, USA) and anti-Asp175 (cleaved caspase 3, 1:100, Cell Signaling Technology, USA) antibodies. Sections were incubated in 3% H_2_O_2_ to block endogenous peroxide activity, and nonspecific binding was blocked with 5% bovine serum in Phosphate Buffered Saline (PBS). Then, sections were incubated with primary antibodies at appropriate dilutions overnight at 4°C, appropriate secondary antibody for 60 min at room temperature, detected with 3,3-diaminobenzidine, and counterstained with hematoxylin. Negative control was added with the normal IgG without the primary antibodies. Positive staining was displayed as brown products. Apoptosis was assessed with a terminal deoxynucleotidyl transferase end-labeling (TUNEL) kit (from Millipore, USA).

Morphological analysis was performed with the Image-Pro Plus v6.0 software (Media Cybernetics, Bethesda, Md). The plaque area was measured as the area between the endothelium and the internal elastic lamina in a cross section. The positive staining area was quantified by a computer-assisted color-gated technique and the ratio of the positive-staining area to plaque area was calculated. Lumen narrowing was defined as the percentage of the plaque area to the area circumscribed by the internal elastic lamina. Maximal lumen narrowing for each plaque was determined by surveying serial sections and the values of maximal lumen narrowing were averaged in each group of mice ^4^. Plaque disruption was defined as a structural defect in the fibrous cap that separates a necrotic core of an atherosclerotic plaque from the lumen, resulting in exposure of the necrotic core to the blood via the gap in the cap ^5^. Plaque disruption was recorded only when the length of discontinuity of the fibrous cap exceeded 200µm in the serial cross-sections. Atherothrombosis was defined as plaque disruption with superimposed thrombus containing platelets and fibrin ^6^. Intraplaque hemorrhage was ascertained by the existence of abundant red blood cells within the plaque ^7^. For calculating the incidence of plaque disruption and atherothrombosis, any disrupted plaque and thrombi in a given animal was counted only once no matter how many sections from one specimen demonstrated evidence of plaque disruption and atherothrombosis.

To examine whether systemic thrombosis and organ infarction may exist, the lung, liver, spleen and kidney of mice in the stress+Ad-ProT group were embedded in paraffin. Serial cross sections of 5 μm in thickness were cut at an interval of 50 μm, and normally 100-400 sections per organ were obtained. All organ sections were stained with H&E staining for histological studies. Histological and morphometric analyses were performed by three independent researchers blindly.

***Western blot***

The protein expression of MMP-2, MMP-9, uncleaved caspase 3 and cleaved caspase 3 was determined by Western blot using specific antibodies. Arteries were lysed with 800 µl lysis buffer (Beyotime, China), and protein was extracted after centrifugation at 14,000 rpm for 10 min (4 ^o^C). Then the samples were heated at 95 ^o^C for 5min in 2 × sample loading buffer and separated by 10% (MMP-2 and MMP-9) or 15% (uncleaved and cleaved caspase 3) SDS-PAGE and electrotransferred to immobilon-P (PVDF) membranes. The membrane was blocked with 5% non-fat milk at room temperature for 2 hrs and then incubated with antibodies specific for MMP-2 (1:800, Abcam), MMP-9 (1:800, Abcam), uncleaved caspase 3 (1:1000, Cell Signaling Technology), cleaved caspase (1:800, Cell Signaling Technology) and β-actin (1:4000, Zhongshan Goldenbridge Biotechnology, China) at 4^o^C for overnight. After three 10 min-washes with TBST, the membranes were incubated with peroxidase-labeled anti-rabbit IgG (1:8000, Dako, USA) at room temperature for 2 hrs. The blots were developed with enhanced chemiluminescence (Millipore).

***PCR array analysis***

Total RNA was extracted from the region proximal to the carotid collar in mice of the non-stress, stress, stress+Ad-ProT and Ad-ProT groups using Trizol reagent (Invitrogen). Reverse transcription was performed in a 20 µl reaction system containing 1.5 µg of total RNA, 500 ng Oligo (dT)_18_ primer, 10 nmol dNTP Mix, and 1 µl superscript III reverse transcriptase (Invitrogen). The cDNA was used for PCR array (Qiagen, SABiosciences, USA) analysis of 84 mouse-specific genes related to atherosclerosis according to the manufacturer’s instructions. The complete list of the genes analyzed is available online at http://www.sabiosciences.com/rt_pcr_product/HTML/PAMM-038A.html. Data analysis was performed with ΔΔCt-based fold-change calculations using a software package provided by the manufacturer.

***Blood coagulation test***

Whole blood was mixed with the acid citrate dextrose solution at a 9:1 ratio, and platelet-poor plasma (PPP) was prepared by centrifugation at 2,000 g for 15 minutes at room temperature followed by a second centrifugation at 10,000 g for 15 min. The concentration of prothrombin was quantified using commercial ELISA kits (ASSAYPRO, USA). The activity of coagulant factor II (prothrombin), VII and VIII, and the parameters of prothrombin time (PT) and activated partial thromboplastin time (APTT) were assessed with clotting assays using reagents for human clotting factor determination in an automated coagulation analyzer (STA-R Evolution, Stago, France).

***Flow cytometry for platelet activation assessment***

Whole blood was mixed with the acid citrate dextrose solution at a 9:1 ratio, and the platelet-rich plasma (PRP) was prepared by centrifugation at 100 g for 15 min. Then the PRP was washed twice with PBS containing 1% FBS and centrifuged at 2000 g for 10 min. Washed platelets were resuspended in PBS to a final concentration of 1×10^7^/ml. Diluted platelet (100 µl) was incubated with FITC-labeled rat anti-CD62P and PE-labeled rat anti-CD41, or relevant isotype control antibodies (BD Phamingen, USA) for 20 min at room temperature. The samples were analyzed using a FACS CALIBUR flow cytometer (BD Biosciences, USA). Particles with positive staining for CD41 were considered to be platelets and those with both CD41^+^ and CD62P^+^ were considered to be activated platelets.

***Plasma corticosterone levels***

Plasma corticosterone levels were measured in non-stress and stressed mice 1 hour after the final stress stimulation, and mice in the stress group 20 hours after the final stress test. As the plasma corticosterone level in mice is stable between 8:00 a.m. and noon ^2^, blood samples were collected during this period of time. The plasma corticosterone levels were quantified using a Corticosterone Enzyme Immunoassay Kit (Assay Designs, AnnArbor, MI, USA) according to the manufacturer’s instruction.

***Plasma lipid profiles***

The plasma levels of total cholesterol, triglycerides, low-density lipoprotein cholesterol, and high-density lipoprotein cholesterol were measured using commercial kits (Roche Diagnostics, Indianapolis, IN).

***Statistical analysis***

Quantitative values were expressed as mean ± standard error of the mean (SEM) and analyzed by unpaired *t*-test or one-way ANOVA as appropriate. In one-way ANOVA analysis, *post hoc* comparisons were carried out with least significant difference test when equal variances were assumed or with Dunnett test when equal variances were not assumed. Qualitative data were analyzed by chi-square test. SPSS 16.0 (SPSS Inc., Chicago, IL, USA) was used for statistical analysis, and a level of *P* < 0.05 was considered significant.

**References**

1. Ni M, et al. Atherosclerotic plaque disruption induced by stress and lipopolysaccharide in apolipoprotein E knockout mice. *Am J Physiol Heart Circ Physiol.* **296**:H1598-H1606 (2009).

2. Bernberg E, Andersson IJ, Tidstrand S, Johansson ME, Bergstrom G. Repeated exposure to stressors do not accelerate atherosclerosis in ApoE-/- mice. *Atherosclerosis.* **204**:90-95 (2009).

3. Schulz C, et al. Effect of chronic treatment with acetylsalicylic acid and clopidogrel on atheroprogression and atherothrombosis in ApoE-deficient mice in vivo. *Thromb Haemost.* **99**:190-195 (2008).

4. von der Thusen JH, van Berkel TJ, Biessen EA. Induction of rapid atherogenesis by perivascular carotid collar placement in apolipoprotein E-deficient and low-density lipoprotein receptor-deficient mice. *Circulation.***103**:1164-1170 (2001).

5. [Falk E](http://www.ncbi.nlm.nih.gov/pubmed?term=Falk E%5BAuthor%5D&cauthor=true&cauthor_uid=17377150), [Schwartz SM](http://www.ncbi.nlm.nih.gov/pubmed?term=Schwartz SM%5BAuthor%5D&cauthor=true&cauthor_uid=17377150), [Galis ZS](http://www.ncbi.nlm.nih.gov/pubmed?term=Galis ZS%5BAuthor%5D&cauthor=true&cauthor_uid=17377150), [Rosenfeld ME](http://www.ncbi.nlm.nih.gov/pubmed?term=Rosenfeld ME%5BAuthor%5D&cauthor=true&cauthor_uid=17377150). Putative murine models of plaque rupture. *[Arterioscler Thromb Vasc Biol](http://www.ncbi.nlm.nih.gov/pubmed?term=Arterioscler Thromb Vasc Biol 2007%3B27%3A969-72" \l "%23)*. **27**:969-972 (2007).

6. [Viles-Gonzalez JF](http://www.ncbi.nlm.nih.gov/pubmed?term=Viles-Gonzalez JF%5BAuthor%5D&cauthor=true&cauthor_uid=15246637), [Fuster V](http://www.ncbi.nlm.nih.gov/pubmed?term=Fuster V%5BAuthor%5D&cauthor=true&cauthor_uid=15246637), [Badimon JJ](http://www.ncbi.nlm.nih.gov/pubmed?term=Badimon JJ%5BAuthor%5D&cauthor=true&cauthor_uid=15246637). Atherothrombosis: a widespread disease with unpredictable and life-threatening consequences. *[Eur Heart J](http://www.ncbi.nlm.nih.gov/pubmed?term=Atherothrombosis%3A A widespread disease with unpredictable and life " \l "%23)*. **25**:1197-1207 (2004).

7. Levy AP, Moreno PR. Intraplaque hemorrhage. *Curr Mol Med* **6**:479-488 (2006).

**Supplementary Figures and Figure Legends**

**
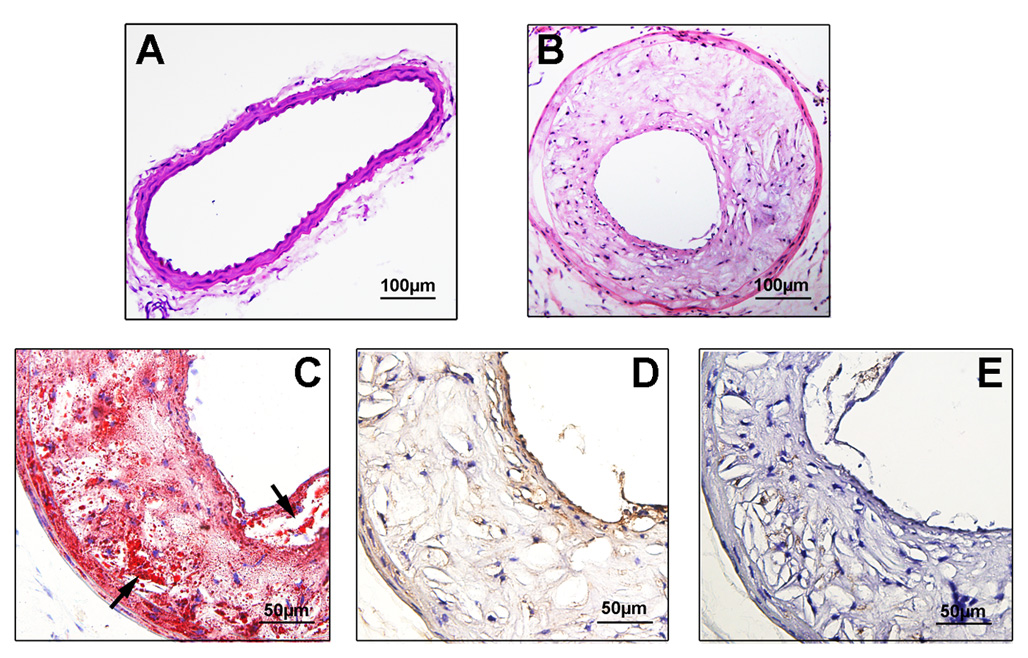
**

**Supplementary Fig. S1. The advanced plaques induced by collar placement for 14 weeks in the right carotid arteries.** (A) Cross-sectional view of the non-collared left carotid artery by H&E staining; (B) Cross-sectional view of the collared right carotid arteries by H&E staining; (C-E) Lipids stained with oil Red O (C), and immunohistochemical stainning for smooth muscle cells (D, α-SMA, brown) and macrophages (E, MOMA-2, brown) for the collar-induced plaques in the right carotid artery. The plaque induced by collar placement around the right carotid artery had scanty cells, but abundant lipids, rich amorphous substance, and a large necrotic core (arrows in C)**.**

**
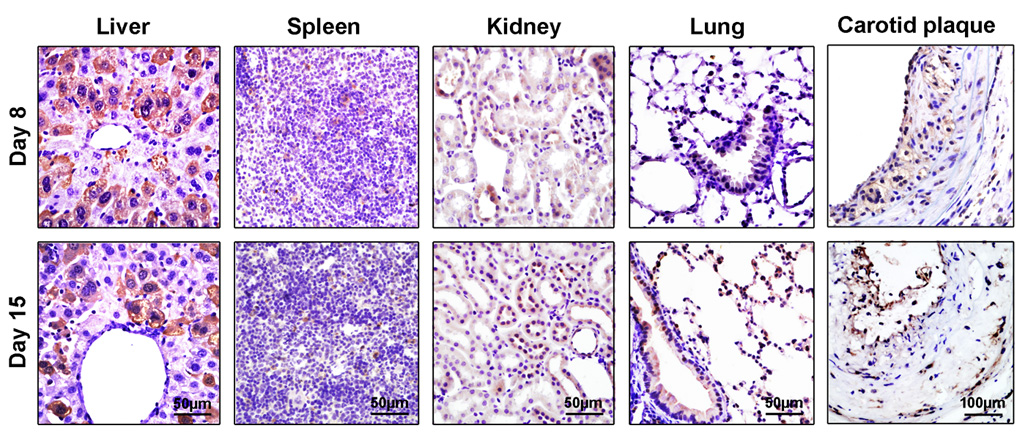
**

**Supplementary Fig. S2. Efficiency of adenovirus transfection *in vivo*.**

Efficiency of adenovirus transfection was assessed by GFP expression using specific antibody. GFP (brown) was rich in the liver, and detectable in the spleen, kidney, lung, and carotid plaques at day 8 (upper panel) and day 15 (lower panel) after adenovirus injection, respectively.

**
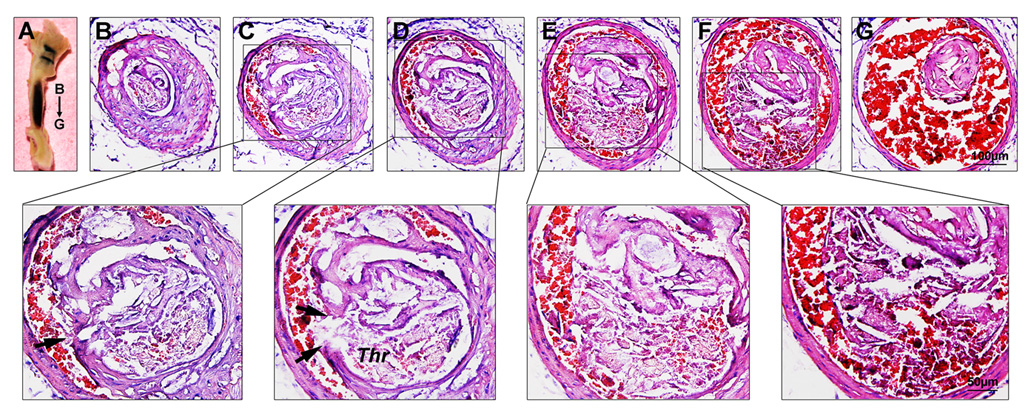
**

**Supplementary Fig. S3. Extensive intraplaque hemorrhage due to plaque disruption in carotid arteries in stress+ Ad-ProT mice.** (A) Gross appearance of severe carotid plaque hemorrhage (the dark-red hematoma-like lesion). (B-G) Serial cross-sections of the intraplaque hemorrhage from the rostral to the caudal end. The bright red areas were blood pools. Intraplaque hemorrhage was associated with plaque disruption (C and D, arrows) and thrombosis (D, Thr), indicating that hemorrhage originated from the lumen via the aperture of disrupted plaque. Extensive intraplaque hemorrhage led to plaque dilapidation (E, F) and vascular expansion (F, G).

**
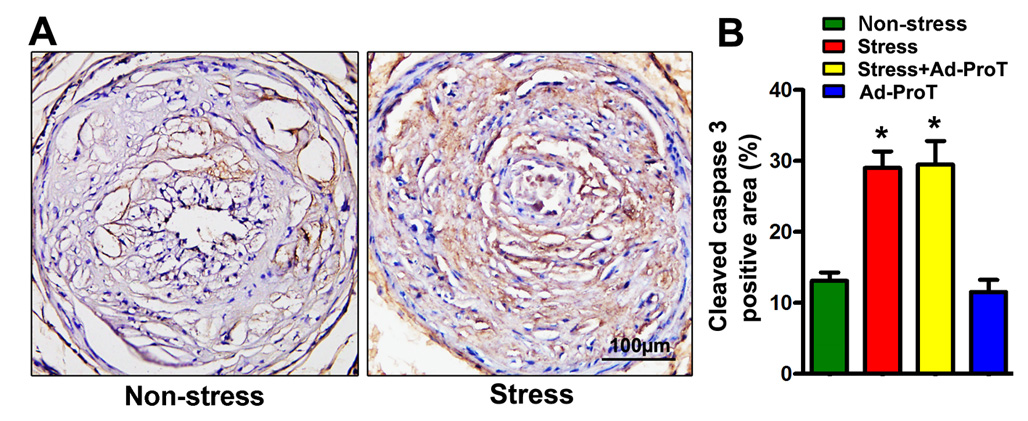
**

**Supplementary Fig. S4. Apoptosis levels in the carotid plaques in the non-stress and stress groups.** (A) Representative immunohistochemistry images for cleaved caspase 3 (brown color) in carotid plaques. (B) Quantitative data of cleaved caspase 3 immunohistochemistry in non-stress, stress, stress+Ad-ProT and Ad-ProT groups. **P* < 0.05 vs. non-stress group (n = 8 each).

**
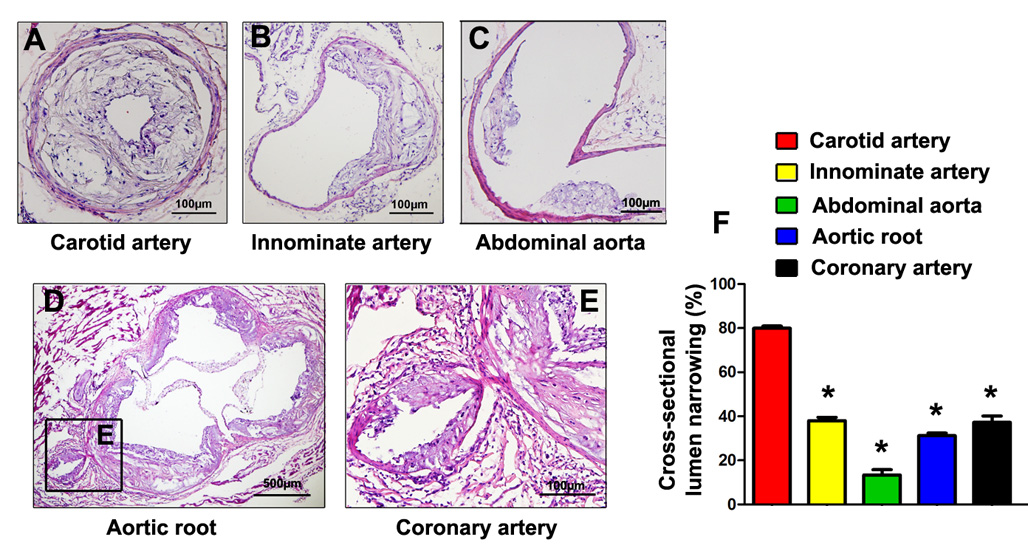
**

**Supplementary Fig. S5. Cross-sectional lumen narrowing of plaques in different** **arteries of mice in the stress+Ad-ProT group.** A-E. Cross-sectional view of plaques in the carotid artery, innominate artery, abdominal aorta, aortic root and coronary artery, respectively, by H&E staining. F. Quantitative analysis of the cross-sectional lumen narrowing of plaques in different arteries of mice. Data were mean ± SEM. * *P* < 0.05 *vs.* plaque in the carotid arteries, *n* = 30.

**Supplementary Tables**

**Supplementary Table S1. Acute and chronic systemic effects of stress treatment** in mice

| Animals | HR  (beats/min) | SBP  (mmHg) | DBP  (mmHg) | Corticosterone levels (ng/ml) |
| --- | --- | --- | --- | --- |
| Non-stress | 453 ± 10 | 108 ± 2 | 88 ± 3 | 26.81 ± 4.06 |
| Stress-treated  (1 hr after stress) | 621 ± 8* | 148 ± 3* | 99 ± 1* | 107.00 ± 7.62* |
| Stress-treated  (20hrs after stress) | 533 ± 13* | 109 ± 1 | 86 ± 2 | 31.45 ± 2.64 |

Values were expressed as mean ± SEM. HR: heart rate; SBP: systolic blood pressure; DBP: diastolic blood pressure; For HR, SBP and DBP measurements, n=14 in each group; For corticosterone, n=7 in each group; **P* < 0.05 *vs.* non-stress mice

**Supplementary Table S2**. Body weight and plasma lipid levels in four groups of mice

| Parameters | Non-stress  (*n* = 14) | Stress  (*n* = 14) | Stress+Ad-ProT  (*n* = 14) | Ad-ProT  (*n* = 14) |
| --- | --- | --- | --- | --- |
| Body weight (g) | 27.06 ± 0.54 | 24.01 ± 0.32 *^†^ | 25.10 ± 0.30*^†^ | 27.06 ± 0.64 |
| Total cholesterol (mmol/L) | 10.85 ± 0.31 | 11.45 ± 0.29 | 11.31 ± 0.27 | 11.30 ± 0.34 |
| Triglycerides (mmol/L) | 0.38 ± 0.02 | 0.32 ± 0.02 | 0.32 ± 0.02 | 0.34 ± 0.02 |
| LDL-C (mmol/L) | 7.94 ± 0.29 | 8.24 ± 0.28 | 7.61 ± 0.35 | 7.90 ± 0.33 |
| HDL-C (mmol/L) | 4.21 ± 0.17 | 4.24 ± 0.18 | 4.26 ± 0.22 | 3.66 ± 0.18 |

Values were expressed as mean ± SEM. Ad-ProT, adenovirus expressing murine prothrombin; LDL-C, low-density lipoprotein cholesterol; HDL-C, high-density lipoprotein cholesterol. * *P* < 0.05 *vs.* non-stress group; ^†^ *P* < 0.05 *vs.* Ad-ProT group.

**Supplementary Table S3. Expression levels of the atherosclerosis-related genes included in the PCR array**

| Genes | Description | Non-stress | Stress | Stress+Ad-ProT | Ad-ProT |
| --- | --- | --- | --- | --- | --- |
| Abca1 | ATP-binding cassette, sub-family A (ABC1), member 1 | 1.00 | 1.49±0.15 | 1.42±0.19 | 1.69±0.23 |
| Ace | Angiotensin I converting enzyme | 1.00 | 0.89±0.15 | 0.84±0.06 | 0.63±0.11 |
| Adfp | Adipose differentiation related protein | 1.00 | 0.77±0.07 | 0.60±0.45 | 0.95±0.35 |
| Apoa1 | Apolipoprotein A-I | 1.00 | 0.66±0.07 | 0.79±0.13 | 1.17±0.26 |
| Apob | Apolipoprotein B | 1.00 | 1.36±0.21 | 1.38±0.17 | 1.30±0.19 |
| Apoe | Apolipoprotein E | 1.00 | 1.19±0.22 | 0.98±0.20 | 1.57±0.13 |
| Bax | Bcl2-associated X protein | 1.00 | 1.06±0.12 | 1.15±0.07 | 1.27±0.29 |
| Bcl2 | B-cell leukemia/lymphoma 2 | 1.00 | 0.45±0.16* | 0.44±0.13* | 0.91±0.18 |
| Bcl2a1a | B-cell leukemia/lymphoma 2 related protein A1a | 1.00 | 1.04±0.13 | 0.98±0.22 | 0.81±0.14 |
| Bcl2l1 | Bcl2-like 1 | 1.00 | 1.13±0.13 | 1.21±0.07 | 1.01±0.26 |
| Bid | BH3 interacting domain death agonist | 1.00 | 0.62±0.13 | 0.85±0.24 | 0.92±0.31 |
| Birc3 | Baculoviral IAP repeat-containing 3 | 1.00 | 1.28±0.30 | 1.56±0.46 | 2.05±0.70 |
| Ccl2 | Chemokine (C-C motif) ligand 2, MCP-1 | 1.00 | 2.26±0.24* | 3.60±0.60* | 2.49±0.33* |
| Ccl5 | Chemokine (C-C motif) ligand 5 | 1.00 | 1.29±0.43 | 1.45±0.55 | 4.18±2.07 |
| Ccr1 | Chemokine (C-C motif) receptor 1 | 1.00 | 2.39±0.19* | 2.54±0.19* | 1.94±0.38 |
| Ccr2 | Chemokine (C-C motif) receptor 2 | 1.00 | 1.10±0.09 | 1.29±0.21 | 0.71±0.15 |
| Cd44 | CD44 antigen | 1.00 | 1.45±0.20 | 1.57±0.34 | 1.44±0.29 |
| Cdh5 | Cadherin 5 | 1.00 | 0.89±0.084 | 0.89±0.09 | 0.79±0.13 |
| Cflar | CASP8 and FADD-like apoptosis regulator | 1.00 | 0.85±0.18 | 0.82±0.12 | 0.64±0.21 |
| Col3a1 | Collagen, type III, alpha 1 | 1.00 | 1.29±0.16 | 1.03±0.23 | 1.00±0.21 |
| Csf2 | Colony stimulating factor 2 (granulocyte-macrophage) | 1.00 | 0.89±0.11 | 1.35±0.20 | 1.17±0.23 |
| Ctgf | Connective tissue growth factor | 1.00 | 0.84±0.12 | 1.17±0.33 | 0.94±0.21 |
| Cxcl1 | Chemokine (C-X-C motif) ligand 1 | 1.00 | 1.67±0.22* | 1.84±0.30* | 1.17±0.22 |
| Eln | Elastin | 1.00 | 0.61±0.08 | 0.56±0.13 | 0.82±0.24 |
| Eng | Endoglin | 1.00 | 1.66±0.16 | 1.27±0.16 | 1.11±0.21 |
| Fabp3 | Fatty acid binding protein 3, muscle and heart | 1.00 | 1.54±0.18 | 1.67±0.25 | 1.46±0.28 |
| Fas | Fas (TNF receptor superfamily member 6) | 1.00 | 0.76±0.06 | 0.85±0.14 | 1.04±0.18 |
| Fga | Fibrinogen alpha chain | 1.00 | 1.00±0.10 | 0.83±0.14 | 0.94±0.15 |
| Fgb | Fibrinogen beta chain | 1.00 | 1.13±0.21 | 1.15±0.16 | 1.35±0.54 |
| Fgf2 | Fibroblast growth factor 2 | 1.00 | 0.44±0.06 | 0.37±0.36 | 0.52±0.13 |
| Fn1 | Fibronectin 1 | 1.00 | 0.52±0.06 | 0.47±0.07 | 0.54±0.13 |
| Hbegf | Heparin-binding EGF-like growth factor | 1.00 | 0.48±0.09 | 0.64±0.09 | 0.83±0.12 |
| Icam1 | Intercellular adhesion molecule 1 | 1.00 | 1.09±0.26 | 1.53±0.43 | 0.98±0.34 |
| Ifng | Interferon gamma | 1.00 | 1.15±0.42 | 4.78±2.67 | 3.34±1.21 |
| Il1a | Interleukin 1 alpha | 1.00 | 2.68±0.66* | 3.28±0.47* | 2.22±0.25* |
| Il1b | Interleukin 1 beta | 1.00 | 2.30±0.31* | 2.16±0.23* | 1.25±0.21 |
| Il1r1 | Interleukin 1 receptor, type I | 1.00 | 1.76±0.25* | 1.55±0.22 | 1.27±0.20 |
| Il1r2 | Interleukin 1 receptor, type II | 1.00 | 3.06±0.33* | 3.67±0.60* | 1.74±0.33 |
| Il2 | Interleukin 2 | 1.00 | 0.92±0.36 | 1.63±0.54 | 1.99±0.79 |
| Il3 | Interleukin 3 | 1.00 | 4.58±0.45* | 4.12±0.65* | 1.28±0.21 |
| Il4 | Interleukin 4 | 1.00 | 1.13±0.17 | 1.46±0.16 | 0.81±0.24 |
| Il5 | Interleukin 5 | 1.00 | 0.94±0.13 | 0.94±0.34 | 1.67±0.98 |
| Itga2 | Integrin alpha 2 | 1.00 | 0.83±0.15 | 0.66±0.05 | 0.86±0.15 |
| Itga5 | Integrin alpha 5 (fibronectin receptor alpha) | 1.00 | 1.09±0.12 | 1.29±0.18 | 0.86±0.16 |
| Itgax | Integrin alpha X | 1.00 | 1.52±0.25 | 1.68±0.26 | 1.30±0.30 |
| Itgb2 | Integrin beta 2 | 1.00 | 2.30±0.16* | 2.48±0.20* | 2.18±0.25* |
| Kdr | Kinase insert domain protein receptor, VEGFR, VEGFR2 | 1.00 | 0.54±0.07* | 0.37±0.11* | 0.42±0.16 |
| Klf2 | Kruppel-like factor 2 (lung) | 1.00 | 0.87±0.10 | 1.10±0.09 | 1.08±0.06 |
| Lama1 | Laminin, alpha 1 | 1.00 | 2.62±0.30* | 3.64±0.43* | 2.05±0.51 |
| Ldlr | Low density lipoprotein receptor | 1.00 | 0.70±0.22 | 0.73±0.10 | 2.36±1.01 |
| Lif | Leukemia inhibitory factor | 1.00 | 0.72±0.08 | 1.27±0.29 | 0.87±0.12 |
| Lpl | Lipoprotein lipase | 1.00 | 1.45±0.16 | 0.80±0.11 | 1.35±0.22 |
| Lypla1 | Lysophospholipase 1 | 1.00 | 0.69±0.05 | 0.52±0.04 | 0.93±0.15 |
| Mmp1a | Matrix metallopeptidase 1a (interstitial collagenase) | 1.00 | 13.41±3.40* | 27.55±6.04* | 3.25±0.54 |
| Mmp3 | Matrix metallopeptidase 3 | 1.00 | 1.29±0.21 | 1.52±0.31 | 0.80±0.19 |
| Msr1 | Macrophage scavenger receptor 1 | 1.00 | 2.45±0.31* | 2.25±0.20* | 1.44±0.33 |
| Nfkb1 | Nuclear factor of kappa light polypeptide gene enhancer in B-cells 1, p105 | 1.00 | 0.77±0.08 | 1.18±0.25 | 1.15±0.29 |
| Npy | Neuropeptide Y | 1.00 | 0.23±0.07* | 0.39±0.14* | 1.02±0.34 |
| Nr1h3 | Nuclear receptor subfamily 1, group H, member 3 | 1.00 | 0.94±0.09 | 1.04±0.11 | 1.07±0.17 |
| Pdgfa | Platelet derived growth factor, alpha | 1.00 | 0.86±0.10 | 0.78±0.12 | 0.76±0.09 |
| Pdgfb | Platelet derived growth factor, B polypeptide | 1.00 | 1.32±0.18 | 1.01±0.09 | 1.33±0.22 |
| Pdgfrb | Platelet derived growth factor receptor, beta polypeptide | 1.00 | 0.83±0.06 | 0.84±0.08 | 0.95±0.08 |
| Ppara | Peroxisome proliferator activated receptor alpha | 1.00 | 0.99±0.24 | 1.22±0.17 | 0.53±0.14 |
| Ppard | Peroxisome proliferator activator receptor delta | 1.00 | 1.08±0.11 | 1.06V0.05 | 1.26±0.22 |
| Pparg | Peroxisome proliferator activated receptor gamma | 1.00 | 1.01±0.14 | 0.66±0.06 | 0.85±0.14 |
| Ptgs1 | Prostaglandin-endoperoxide synthase 1 | 1.00 | 1.18±0.15 | 1.16±0.09 | 0.95±0.21 |
| Rxra | Retinoid X receptor alpha | 1.00 | 1.17±0.12 | 1.15±0.16 | 1.35±0.33 |
| Sele | Selectin, endothelial cell; E-selectin | 1.00 | 2.71±0.30* | 2.32±0.24* | 2.20±0.41 |
| Sell | Selectin, lymphocyte; L-selectin | 1.00 | 1.45±0.58 | 2.36±1.08 | 1.59±1.00 |
| Selp | Selectin, platelet; P-selectin | 1.00 | 1.12±0.19 | 1.41±0.16 | 0.90±0.19 |
| Selplg | Selectin, platelet (p-selectin) ligand | 1.00 | 1.10±0.13 | 1.84±0.23 | 1.87±0.55 |
| Serpinb2 | Serine (or cysteine) peptidase inhibitor, clade B, member 2; PAI-2 | 1.00 | 0.80±0.11 | 1.16±0.07 | 0.89±0.28 |
| Serpine1 | Serine (or cysteine) peptidase inhibitor, clade E, member 1; PAI-1 | 1.00 | 0.66±0.09 | 0.84±0.08 | 0.87±0.15 |
| Sod1 | Superoxide dismutase 1, soluble | 1.00 | 0.84±0.12 | 0.75±0.11 | 0.76±0.15 |
| Spp1 | Secreted phosphoprotein 1;Opn | 1.00 | 3.42±0.56* | 3.41±0.29* | 3.66±1.03* |
| Tgfb1 | Transforming growth factor, beta 1 | 1.00 | 0.51±0.05* | 0.53±0.05* | 0.23±0.08* |
| Tgfb2 | Transforming growth factor, beta 2 | 1.00 | 0.81±0.09 | 0.99±0.11 | 0.79±0.17 |
| Thbs4 | Thrombospondin 4 | 1.00 | 0.87±0.07 | 1.03±0.06 | 1.43±0.34 |
| Tnc | Tenascin C | 1.00 | 0.97±0.08 | 0.77±0.11 | 1.03±0.04 |
| Tnf | Tumor necrosis factor | 1.00 | 1.61±0.29 | 2.97±1.27 | 2.24±0.96 |
| Tnfaip3 | Tumor necrosis factor, alpha-induced protein 3 | 1.00 | 0.42±0.05* | 0.40±0.06* | 0.51±0.10 |
| Vcam1 | Vascular cell adhesion molecule 1 | 1.00 | 1.52±0.14 | 1.67±0.15 | 1.49±0.21 |
| Vegfa | Vascular endothelial growth factor A | 1.00 | 1.21±0.24 | 0.86±0.07 | 1.37±0.32 |
| Vwf | Von Willebrand factor homolog | 1.00 | 0.52±0.11 | 0.57±0.10 | 0.12±0.03 |

Levels of mRNA expression of the genes were presented as fold of that of the non-stress group. Data were expressed as mean ± SEM, n = 6 for each group. **P* < 0.05, vs. non-stress group.
